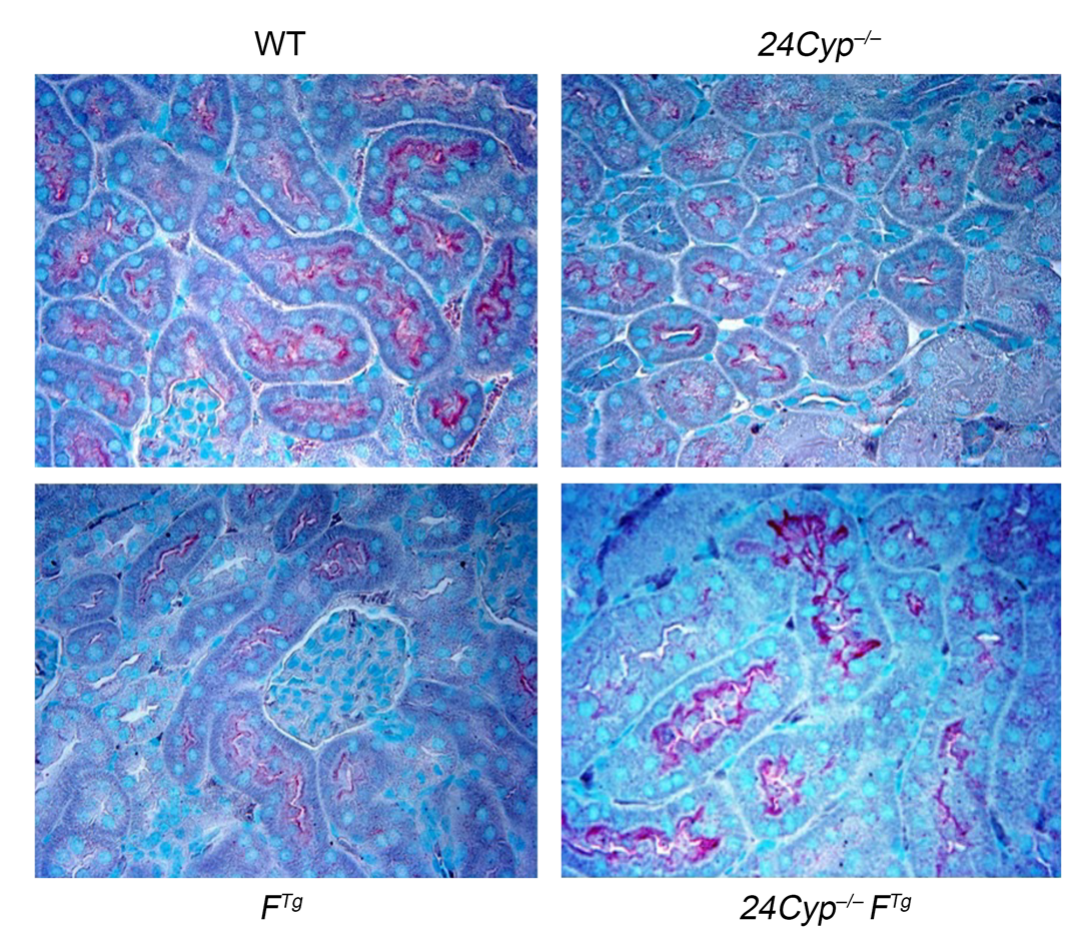# CYP24 inhibition as a therapeutic target in FGF23-mediated renal phosphate wasting disorders

**DOI:** 10.1172/JCI193400

**Published:** 2025-04-15

**Authors:** Xiuying Bai, Dengshun Miao, Sophia Xiao, Dinghong Qiu, René St-Arnaud, Martin Petkovich, Ajay Gupta, David Goltzman, Andrew C. Karaplis

Original citation: *J Clin Invest*. 2016;126(2):667–680. https://doi.org/10.1172/JCI81928

Citation for this corrigendum: *J Clin Invest*. 2025;135(8):e193400. https://doi.org/10.1172/JCI193400

The authors recently became aware that in Figure 4L of the original article, the WT image and the *Cyp24^–/–^F^Tg^* image were different crops of the same image. The correct panel, provided from the original source data, is shown below.

The authors regret the error.

## Figures and Tables

**Figure 4L:**